# Improvement of the Bioavailability and Glycaemic Metabolism of Cinnamon Oil in Rats by Liquid Loadable Tablets

**DOI:** 10.1100/2012/681534

**Published:** 2012-04-01

**Authors:** Chunchao Han, Bo Cui

**Affiliations:** ^1^School of Pharmacy, Shandong University of Traditional Chinese Medicine, Jinan 250355, China; ^2^College of Food and Biological Engineering, Shandong Polytechnic University, Jinan 250353, China

## Abstract

The purpose of this study is to investigate the bioavailability and glycaemic metabolism of cinnamon oil (CIO) carried by liquid-loadable tablets (CIO-LLTs), the carrier of a CIO self-emulsifying formulation (CIO-LS). The results of tests performed to evaluate the physical properties of the CIO-LLT complied with Chinese Pharmacopeia (2010). The release profile suggested that the CIO-LLT preserved the enhancement of in vitro dissolution of cio. After orally administration, the plasma concentration-time profile and pharmacokinetic parameters suggested that a significant increase (*P* < 0.0001) in the *C*_max_, AUC and *F* were observed in the CIO-LLT. The blood glucose and the HbA1c were significantly decreased in alloxan-induced hyperglycemic rats (*P* < 0.05, *P* < 0.01, resp.), while the level of insulin secretion was markedly elevated in alloxan-induced hyperglycemic rats (*P* < 0.05). The alloxan-damaged pancreatic *β*-cells of the rats were partly recovered gradually after the rats were administered with CIO-LLT 45 days later. CIO-LLT could improve the bioavailability and glycaemic metabolism of CIO.

## 1. Introduction

According to the World Health Organization, it is estimated that approximately 150 million people worldwide have diabetes mellitus at present [1]. Therefore, new therapeutic approaches are needed to treat diabetes more efficiently. Presently, there is growing interest in herbal remedies due to the side effects associated with the oral hypoglycemic agents for the treatment of diabetes mellitus [2]. The plant kingdom is a wide field to search for natural effective oral hypoglycemic or hypolipidemic agent that has slight or no side effects. More than 400 plants with glucose lowering effect are known [3]. They might be used in various forms like food and medicines, which contain both organic and inorganic constituents.

Cinnamon is one of the well-known and oldest spices, which has been used for centuries in several cultures [4]. It has been traditionally used in Ayurvedic and Chinese medicine as a treatment for diabetes [5]. Research has shown that cinnamon oil (CIO) is particularly very helpful for patients suffering from type 2 diabetes. However, the poor water-soluble drug, cinnamon oil, leads to poor dissolution and bioavailability. To overcome these problems, various formulation strategies such as cyclodextrin, surfactants, nanoparticles, lipids carriers, and solid dispersions are reported [6]. In recent years, much attention has been focused on self-emulsifying drug delivery systems (SEDDSs), which are isotropic mixtures of oil, surfactant, cosurfactant, and drug that form fine oil-in-water emulsion when introduced into aqueous medium under gentle agitation [7–9]. SEDDSs have shown a reasonable success in improving oral bioavailability of poorly water soluble drugs [10, 11].

 However, SEDDSs are usually formulated in a liquid form which has some disadvantages such as high production costs, low drug incompatibility, and poor physical and chemical stability. The incorporation of the self-emulsifying mixture into a solid dosage form is desirable as it combines the advantages of SEDDS (high solubility and bioavailability) with those of solid dosage forms (high stability). The transformation of liquid lipid systems into a solid oral dosage form has been attempted by several methods such as capsule filling, spray drying, adsorption onto solid carriers, and melt granulation as well as other techniques [12–15]. The tablet is the most popular dosage form in use today. It is simple and convenient to use. Liquid-loadable tablets (LLTs), prepared by direct compression of solid carrier particles and subsequently loading the liquid into the tablet, have a higher liquid loading [16]. The work presented in this paper focuses on the pharmacokinetic (PK) performance of CIO from loaded LLT and the effects of CIO-loadable tablets (CIO-LLT) on glycaemic metabolism.

## 2. Materials and Methods

### 2.1. Animals

Healthy male adult Wistar rats (2 months old and weighing 225 ± 25 g) were used in the study. The study was approved by Shandong University's ethics committee, and all procedures complied with the guidance set out in the Guidelines for Caring for Experimental Animals published by the Ministry of Science and Technology of China. Every care was taken to minimize discomfort, distress, and pain.

### 2.2. Materials

Cinnamon oil was obtained from Shanghai Yansheng biotechnology Co., Ltd. China. Magnesium aluminometasilicate (MAS) was obtained from Jinan Kailida Chemical Technology Co., Ltd China. 

### 2.3. Preparation of CIO-Loaded SEDDS (CIO-LS)

Based on the pilot studies, the blank SEDDSs were prepared by mixing of 30% Miglyol 812 (oil), 60% Cremophor RH40 and Tween 80 (surfactant, 2 : 1), and 10% Transcutol P (cosurfactant) at 50°C with a magnetic stirrer. Then CIO and Aerosil 200 (1000 mg) suspended in 150 mL ethanol were dissolved in the blank SEDDS with stirring until forming an isotropic mixture. The mixture was then kept at room temperature and equilibrating for 24 h.

### 2.4. Reconstitution Properties of the SEDDS

The emulsification time of the SEDDS formulations was evaluated according to the way described in detail by Xi et al. [17]. In brief, SEDDS (250 *μ*L) was introduced into 500 mL of distilled water at 37°C under gentle agitation by a standard stainless steel dissolution paddle rotating at 50 rpm. The emulsification time was assessed visually. All experiments were carried out in triplicates.

Droplet size was determined by Zetasizer Nano ZS (Jinan Runzhi Instruments, China) with dynamic light scattering particle size analyzer at a wavelength of 635 nm and at a scattering angle of 90° at 25°C. All studies were repeated three times, and the values of *z*-average diameters were used. The *z*-average diameter, also referred to as the harmonic intensity-weighted average hydrodynamic diameter, of the emulsions was derived from cumulated analysis by the Automeasure software. Zeta potential of the emulsion formed after addition of SEDDS into 0.1 N HCl solution was measured using Zetasizer Nano ZS.

### 2.5. Preparation of LLT

The composition of LLT was MAS/magnesium stearate (99.5 : 0.5 w/w%)[16]. The additive was required during the tabletting process to avoid undesired adhesion of MAS powder to the tablet punches when under compression. Tablets of approximately 250 mg were compressed using a round 12 mm flat-faced punch. Tablet hardness was determined using a hardness tester. Tablet disintegration was performed in 0.1 N HCl using an automatic disintegration tester (JB-1, Tianjian Xinzhou, China) according to the conditions of the Chinese Pharmacopoeia (2010) for uncoated tablets.

### 2.6. Loading of CIO in to Tablet

Tablets were placed in excess of CIO-SEDDS and allowed to absorb the liquid until a constant tablet weight was reached. Before weighing, excess of liquid on the surface of the tablets was removed by tissue paper. After few hours of storage, the tablet surface is dry, comparable to the nonloaded tablet [18].

### 2.7. Characterization of CIO-Loaded Tablet (CIO-LLT)

Tablets were evaluated by performing quality control tests for uniformity of drug content, friability, disintegration, hardness, and weight variation. All tests were carried out in triplicates and according to Chinese Pharmacopoeia (2010).

### 2.8. In Vitro Drug Release Study

CIO-LS and CIO-LLT were tested in vitro in 950 mL purified water at 37°C using rotating basket apparatus (Chinese Pharmacopoeia) at a rotation speed of 100 rpm. Samples were withdrawn at predetermined time intervals and then mixed with 150 *μ*L of methanol containing 1.75 *μ*g of *α*-naphthoflavone. The resultant solution was filtered, and an aliquot (100 *μ*L) of the filtrate was subjected to HPLC analysis.

### 2.9. In Vivo Protocols

Wistar rats were divided randomly into four groups (six animals in each group). The first group (CIO) was treated orally (100 mg/kg) CIO formulated in normal saline containing 3% Tween 80. Group II (CIO-LS) and group III (CIO-LLT) were treated orally CIO-SEDDS (100 mg CIO/kg) and CIO-LLT (100 mg CIO/kg), respectively. Group IV was treated (i.v.) CIO sample (100 mg/kg) used as control group. Before dosing, the rats were fasted overnight. Food was given minimum 2 h after dosing. A 3 mL blood samples were drawn from the tail vein before dosing and at the following times: 15 min, 30 min, 45 min, 1 h, 1.5 h, 3 h, 4 h, 6 h, and 24 h after dosing. Blood samples were centrifuged at 3000 g for 10 min, and plasma samples were harvested. Each plasma sample (100 *μ*L) was mixed with 200 *μ*L of Methanol, vortexed, and centrifuged at 16,000 g for 5 min. The supernatant was filtered with a 0.45-*μ*m syringe filter, and 100 *μ*L of the filtrate was subjected to HPLC analysis.

### 2.10. Pharmacokinetic Parameter Analyses

Pharmacokinetic parameters were calculated from the corresponding plasma concentration-time curves using noncompartmental analysis (WinNonlin 4.0, Pharsight, Mountain View, CA, USA). The area under the plasma concentration-time curve from time 0 to time infinity (AUC_0–∞_), *C*_max⁡_, *T*_max⁡_ were determined from the concentration-time profile. The absolute bioavailability (*F*) was calculated using the following equations:

(1) $$F=\frac{\left({\text{AUC}}_{\text{oral}/\text{i}.\text{p}.}^{}*{\text{Dose}}_{\text{i}.\text{v}.}\right)}{\left({\text{AUC}}_{\text{i}.\text{v}.}^{}*{\text{Dose}}_{\text{oral}/\text{i}.\text{p}.}\right)}.$$

### 2.11. Estimation of CIO-LLT on Glycaemic Metabolism

Animals were fasted for 12 h and were then injected (i.v.) with alloxan (75 mg/kg) solution that was made with saline. Forty-eight hours later, blood samples were collected from the tail veins of the rats. The blood glucose was analyzed with a Glucometer-4 (Bayer). The blood glucose level of rats greater than 11.1 mmol/L was selected as hyperglycemic rats.

Forty hyperglycemic rats were selected and randomly divided into 4 groups. The first group (CIO) was treated orally (100 mg/kg) CIO formulated in normal saline containing 3% Tween 80. Group II (CIO-LS) and group III (CIO-LLT) were treated orally CIO-SEDDS (100 mg CIO/kg) and CIO-LLT (100 mg CIO/kg), respectively. Group IV was treated orally saline used as control group. On the 45th day, blood samples were collected from the orbital veins to measure the to determine the blood glucose levels and HbA1c with the HbA1c Apparatus (Variant II, Bio-Rad Laboratories) and insulin with an enzyme-linked immunosorbant assay (ELISA) kit (Shanghai Jinma Biological Technology, Inc., China), respectively. Then, the rats were sacrificed. The pancreas was dissected out and placed in 10% buffered formalin and the liver was dissected out for the measurement of hepatic glycogen.

### 2.12. Data Analysis

The data are expressed as mean ± SEM. Statistical significance was tested by two-tailed Student's *t*-test for differences between two groups and by one-way ANOVA carried out using the SAS statistical package (version 8.1, SAS Institute, Cary, NC, USA) for testing differences between means for more than two groups. Statistical significance level was set at *P* < 0.05.

## 3. Results and Discussion

The work presented in this paper focuses on the ability of LLT prepared from MAS to be loaded with a SEDDS system containing CIO, the pharmacokinetic (PK) performance of CIO from loaded LLT and the effects of CIO-loadable tablets (CIO-LLT) on glycaemic metabolism. MAS is nontoxic and consists of highly porous spherical particles with a median size of 110 *μ*m and a specific surface area of approximately 370–420 m^2^/g [16]. Due to its excellent fluidity and compressibility, MAS can be mechanically compacted into stable tablets. The mechanical stability of the unloaded LLT was 29 N ± 8% (*n* = 10). Disintegration time of the unloaded LLT was 0.27 ± 0.11 min. It was the time taken by each of the 6 tablets to pass completely through the 10 mesh screen.

### 3.1. Reconstitution Properties of the SEDDS

SEDDS formulation disperse quickly and completely when subjected to aqueous environment under mild agitation. The efficiency of self-emulsification can be estimated by measuring the rate of emulsification and the droplet size distribution [19]. As shown in Table 1, emulsification time was 23 s for SEDDS. It is much related to their hydrophilic-lipophilic balance (HLB) value of surfactant and cosurfactant.

It has been reported that any change in interfacial film influences the surface curvature of the droplet leading to differences in the droplet size [20–22]. As shown in Table 1, the *z*-average droplet sizes for SEDDS system was 132.5 nm. The mean droplet size and size distribution (polydispersity index) was 0.160. SEDDS showed a zeta potential of +2.00 mV, therefore providing prolonged stabilization.

### 3.2. Loading of LLT

**Figure 1:**
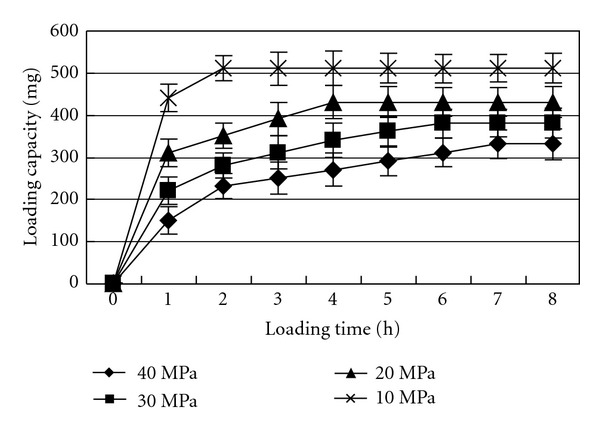
Correlation between SEDDS loading and loading time.

**Figure 2:**
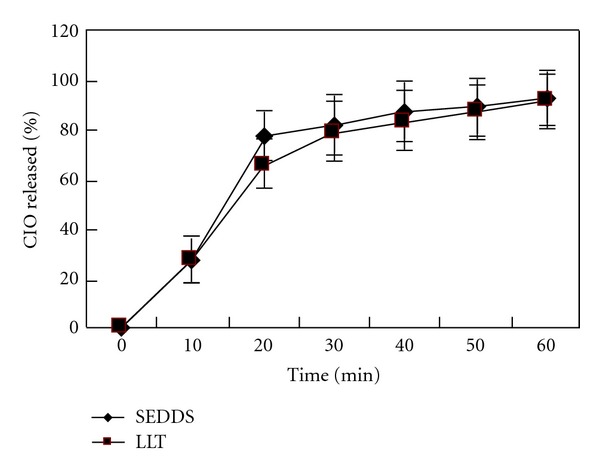
Drug release profiles of the SEDDS and LLT.

A series of absorption experiments was conducted to explore the loading capacity of the LLT.  Figure 2 illustrates the absorption profiles of SEDDS into the LLT as a function of exposure time and the compression force. As shown in Figure 1, application of higher compression force during tablet compression increased the loading time and reduced the maximum theoretical loading amount. The loading process can be seen as the replacement of air in the tablet pores by liquid. It is consistent with the Washburn equation [23]: *v* = *r* ^2^/8 *μ*_eff_∗*p*_*c*_/*d* where *v* is the velocity of the interface between air and liquid, *r* is the radius and *d* the length of a pore, *p*_*c*_ the capillary pressure and *μ*_eff_ is the effective viscosity. According to the equation, the capillary flow in the loading process is controlled mainly by capillary action. The fast absorption is caused by capillary suction and is determined by both the pore size distribution of LLT, the nature of the liquid viscosity and liquid-solid contact angle [16]. The higher compression pressure applied would cause the formation of smaller pore size distribution [18]. It may explain why the loading time increases at higher compression pressures and why the LLT subjected to higher compression pressure absorbed lower theoretical amount of SEDDS. 

### 3.3. Characterization of CIO-Loaded Tablet

Table 2 shows the results of tests performed to evaluate the physical properties of the CIO-loaded tablet according to Chinese Pharmacopoeia (2010). It is clear that CIO-loaded tablet complied with the required specifications and standard regarding drug content uniformity. CIO-loaded tablet complied with Chinese Pharmacopeia friability test as the friability was less than 1% and there were no broken tablets. For the disintegration time results, CIO-loaded tablet showed very short disintegration time (0.31 ± 0.20 seconds). Also, the CIO-loaded tablet passed the Chinese Pharmacopeia weight variation test.

### 3.4. In Vitro Drug Release Study

Drug release rate and the cumulative percent of CIO dissolved into the aqueous medium are important criteria that govern the quality of the LLT. The physical properties of the ingredients used to prepare the LLT have a profound effect on the CIO release rate. Drug release profiles of the CIO-tablet and CIO-SEDDS are shown in Figure 2. The CIO dissolution from both SEDDS and LLT formulations took place immediately. The fast drug release of the LLT may be explained by both an increase in the specific surface area of the drug resulting from the adsorption to the highly MAS porous spherical particles and possibly an amorphous state of the drug. The solid carrier, MAS, used in the LLT did not interfere the dissolution of CIO from the SEDDS. The results of the release profile suggested that the LLT preserved the enhancement of in vitro dissolution of CIO and would eventually enhance the dissolution of drug in vivo.

### 3.5. Pharmacokinetics of CIO-Loaded Tablet

**Figure 3:**
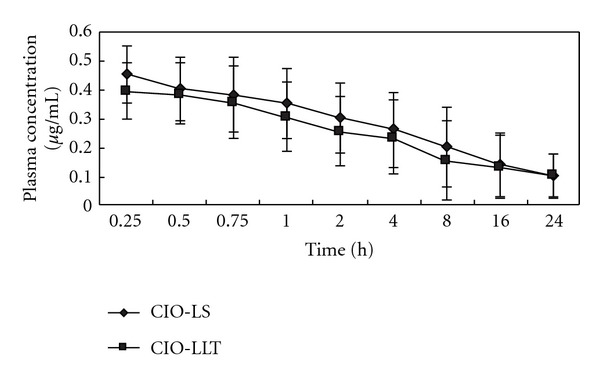
Mean whole blood concentrations of CIO in rats following oral doses of 100 mg in the form of CIO-SEDDS and CIO- LLT. The total plasma concentrations of drug after oral administration of CIO could not be detected.

Figure 3 shows plasma concentration-time curves of CIO after administration of three different formulations. The calculated pharmacokinetic parameters are summarized in Table 3. After orally administration, only the plasma concentration-time profile and pharmacokinetic parameters of SEDDS and LLT could be obtained at the dose of 100 mg/kg. The data of CIO were not meaningful because plasma levels of CIO were either below detectable limit and they were not high enough for accurate pharmacokinetic analysis. In the case of SEDDS (CI-LS), CIO was rapidly absorbed (*T*_max⁡_, 0.15 ± 0.09 h) reaching a *C*_max⁡_ of 0.51 ± 0.30 *μ*g/mL. In the case of LLT (CIO-LLT), CIO was absorbed (*T*_max⁡_, 0.18 ± 0.12 h) reaching a *C*_max⁡_ of 0.43 ± 0.21 *μ*g/mL. The bioavailability of CIO from each of the 3 formulations, estimated as the area under the whole blood curve was 0, 2.5% and 2.2%, respectively. It was compared using two-tailed Student's *t*-test. It did not show any statistically significant differences between the CIO-LS and CIO-LLT.

 A significant increase (*P* < 0.0001) in the *C*_max⁡_, AUC and *F* were observed in the CIO-LLT group when compared with the CIO group. Thus, the higher plasma concentrations of CIO in LLT were contributed by improving the solubility of drug by SEDDS.

### 3.6. Effects of CIO-LLT on Glycaemic Metabolism

Alloxan is the most prominent diabetogenic chemicals in diabetes research. It is toxic glucose analogues that preferentially accumulate in pancreatic beta cells via the GLUT2 glucose transporter [24, 25]. Alloxan has two distinct pathological effects: it selectively inhibits glucose-induced insulin secretion through specific inhibition of glucokinase, the glucose sensor of the beta cell, and it causes a state of insulin-dependent diabetes by selective necrosis of beta cells in type 1 and type 2 diabetes mellitus [24, 25]. So alloxan is the agent of choice for induction of diabetic experimental animals in this study.

The results of blood glucose from hyperglycemic rats induced by alloxan are presented in Table 4. The levels of blood glucose decreased after administration of CIO-LS and CIO-LLT (*P* < 0.05). CIO-LS and CIO-LLT could decrease the concentration of HbA1c in plasma of alloxan-induced hyperglycemic group 45 days later (*P* < 0.01), as shown in Table 4. However the same result did not occur in the CIO group.

**Figure 4:**
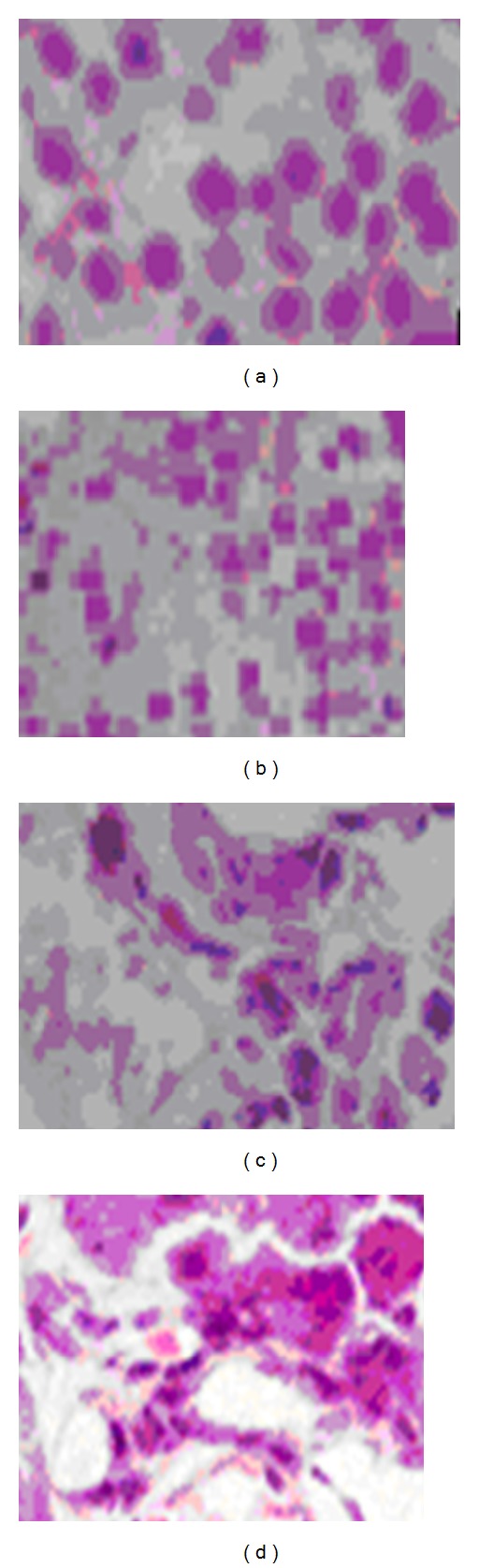
Islet cell death and replication represented by hematoxylin-eosin.

The mechanisms of the hypoglycaemic effect of CIO-LS and CIO-LLT have been also studied in this paper. As shown in Table 5, the levels of serum insulin elevated after administration of CIO-LS and CIO-LLT. However, the same results did not occur in the saline treated group (3.8 ± 0.4 *μ*U/mL) throughout the total duration of the study. It is possible that CIO bring about release of insulin from the surviving *β*-cells, as well from the recovered *β*-cells by CIO. The *β*-cells of the rats fed with CIO-LS and CIO-LLT were partly recovered (Figures 4(a) and 4(b)). *β*-Cells death and alteration of islet cell population were prominent in the diabetic rats that fed with CIO (Figure 4(c)) and saline (Figure 4(d)).

Glycogen storage in the liver is another way to maintain blood glucose concentration in mammals. Decreased hepatic glucose production is induced by glycogen synthesis. CIO-LS and CIO-LLT produced the increase in the level of hepatic glycogen. Concentrations of hepatic glycogen were lower in saline-treated rats than CIO-treated rats (Table 5). However, there is no significant difference between them (*P* > 0.05).

## 4. Conclusion

The work presented in this paper focuses on the ability of LLT prepared from MAS to be loaded with a SEDDS system containing CIO. It combines the advantages of SEDDS (high solubility and bioavailability) with those of solid dosage forms (high stability). CIO-loaded tablet could improve the glycaemic metabolism of CIO. It was contributed by improving the bioavailability drug by SEDDS.

## Figures and Tables

**Table 1:** Reconstitution properties of the SEDDS.

	SEDDS
Emulsification time (seconds)	23 ± 3
Zeta potential	+2.00 ± 0.30
Droplet size (nm)	132.5
Polydispersity index (PDI)	0.160 ± 0.020

Values are shown as means ± SD.

**Table 2:** Characteristics of the CIO-loaded tablet.

	CIO-loaded tablet
Average CIO contents (% ± SD)	96.11 ± 1.5
Friability (% ± SD)	0.60 ± 0.18
Hardness (N ± SD)	30.1 ± 7.00
Disintegration time (min ± SD)	0.31 ± 0.20
Weight variation (mg)	1.55 ± 0.022

**Table 3:** Pharmacokinetic parameters after oral administration of CIO, CIO-LS, and CIO-LLT to rats.

Groups	AUC_0→24 h_ (*μ*g h/mL)	*C*_max⁡_ (*μ*g/mL)	*T*_max⁡_ (h)	*F* (%)
CIO-LS	1.96	0.51 ± 0.30	0.15 ± 0.09	2.5
CIO-LLT	1.68	0.43 ± 0.21	0.18 ± 0.12	2.2
CIO	NA	NA	NA	0

Values are shown as means ± SD. *P < 0.0001, NA: Not applicable. (*n* = 6).

**Table 4:** Effect of CIO, CIO-LS and CIO-LLT on blood glucose and HbA1c levels in alloxan-hyperglycemic rats.

Different groups	Blood glucose (mmol/L)	HbA1c
Control group	21.2 ± 2.1	11.8 ± 0.23
CIO-LS-treated	10.4 ± 3.0*	7.0 ± 0.31**
CIO-LLT-treated	12.5 ± 2.1*	7.9 ± 0.20**
CIO-treated	19.9 ± 2.7	11.0 ± 0.20

Values are means ± SEM, *n* = 10. *P < 0.05, **P < 0.01 versus Control group.

**Table 5:** Effect of CIO, CIO-LS and CIO-LLT on serum insulin and hepatic glycogen level in alloxan-induced diabetic rats.

Different groups	Serum insulin (*μ*U/mL)	Hepatic glycogen (mg/g tissue)
Control group	3.9 ± 1.4	13.3 ± 3.6
CIO-LS-treated	8.9 ± 0.7**	17.6 ± 0.5*
CIO-LLT-treated	6.8 ± 0.5**	16.8 ± 0.6*
CIO-treated	4.3 ± 0.4	13.8 ± 0.5

Values are means ± SEM, *n* = 10. *P < 0.05, **P < 0.01 versus Control group.
